# Diagnosis of Operating Conditions of the Electrical Submersible Pump via Machine Learning

**DOI:** 10.3390/s23010279

**Published:** 2022-12-27

**Authors:** Jéssica Brasil, Carla Maitelli, João Nascimento, Osvaldo Chiavone-Filho, Edney Galvão

**Affiliations:** 1 Department of Chemical Engineering (DEQ), Federal University of Rio Grande do Norte (UFRN), Natal 59078-970, Brazil; 2 Department of Petroleum Engineering (DPET), Federal University of Rio Grande do Norte (UFRN), Natal 59078-970, Brazil; 3 Federal Institute of Education, Science and Technology of Rio Grande do Norte (IFRN), Parnamirim 59143-455, Brazil

**Keywords:** artificial lift, electrical submersible pump, machine learning, classification algorithms

## Abstract

In wells that operate by electrical submersible pump (ESP), the use of automation tools becomes essential in the interpretation of data. However, the fact that the wells work with automated systems does not guarantee the early diagnosis of operating conditions. The analysis of amperimetric charts is one of the ways to identify fail conditions. Generally, the analysis of these histographics is performed by operators who are often overloaded, generating a decrease in the efficiency of observing the well operating conditions. Currently, technologies based on machine learning (ML) algorithms create solutions to early diagnose abnormalities in the well’s operation. Thus, this work aims to provide a proposal for detecting the operating conditions of the ESP pump from electrical current data from 24 wells in the city of Mossoró, Rio Grande do Norte state, Brazil. The algorithms used were Decision Tree, Support Vector Machine, K-Nearest Neighbor and Neural Network. The algorithms were tested without and with hyperparameter tuning based on a training dataset. The results confirm that the application of the ML algorithm is feasible for classifying the operating conditions of the ESP pump, as all had an accuracy greater than 87%, with the best result being the application of the SVM model, which reached an accuracy of 93%.

## 1. Introduction

Over the decades, the production system has been modified due to population growth and the growing demand for inputs that provide longevity and quality to the life of the planet’s inhabitants. Sectors such as telecommunications, health, and transport have benefited significantly and quickly through the use of technologies that connect more and more people. Moreover, it can help in the agility of decision-making in corporate groups.

The notoriety in the use of techniques and topics related to Industry 4.0 is gaining more prominence in the oil and gas sector. This sector has witnessed great advances due to the adoption of the concepts and technologies of the digital field. These technologies empower oil and gas facilities with detection equipment to enable real-time monitoring of wells and other assets in an oil field. The existence of such tools with digital world connection is essential for the industry [1].

In artificial lift, the use of electrical submersible pump (ESP) has been growing in the oil industry as it is an effective and economical method. This artificial lift method helps to maximize the production of recoverable oil, achieving higher withdrawals and handling of a greater volume of fluid [2]. The lifespan of the ESP is affected by repeated shutdowns. These sudden failures can be generated by poor management and design conditions not being specific to the artificial lift system [3].

The ESP system comprises the electric motor, protector, gas separator or intake, multi-stage centrifugal pump, power cable, motor, transformer, and a reliable power source. Downhole sensors or gauges are installed to monitor the well and pump operating conditions [4].

**Figure 1:**
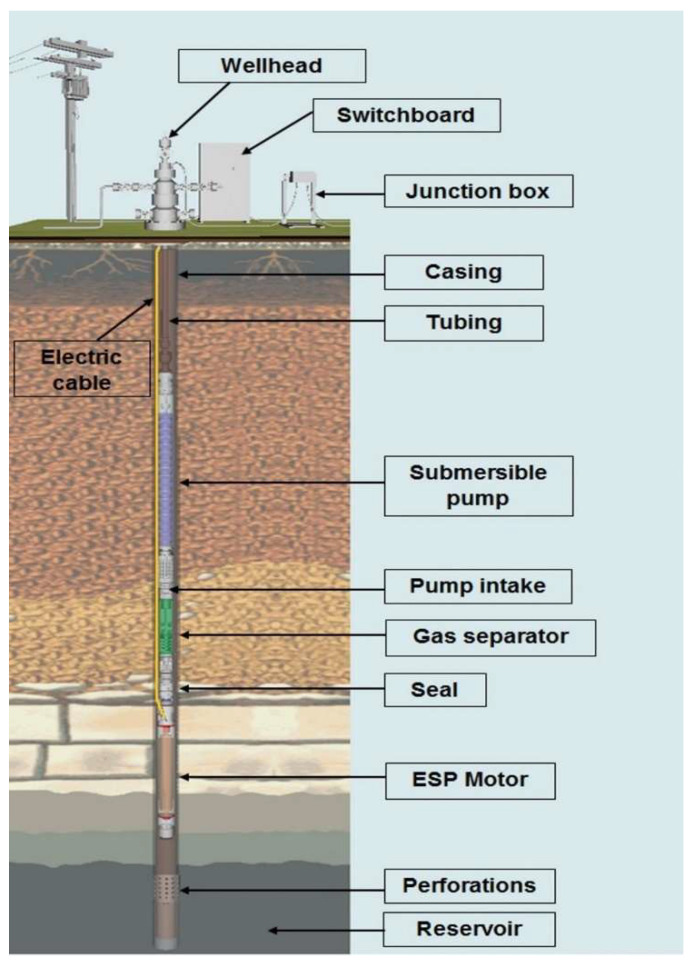
Onshore ESP system [6].

The electric motor is of the three-phase type and works with an alternating electric current. The motor is driven from the electrical cable that leaves the surface to the bottom of the production column, where the motor is positioned. The protector allows the contraction and expansion of the engine’s insulating oil, ensuring no contamination by well fluid. The gas separator ensures that fluid enters the pump’s first stage and removes small amounts of gas. The multistage pump is responsible for pumping the fluids. The wellhead ensures the passage of the electrical cable to the bottom of the well and supports the weight of the string. The junction box connects the surface and bottom cables. The switchboard contains load protection and control equipment [5]. This system can be seen in Figure 1.

The ESP systems can be installed in urban areas and the most equipment of the ESP is in the subsurface; takes up little space, so it is very suitable for offshore environments. It is ideal for high flow rates of liquid, requiring low maintenance. It can be used in deviated wells, and corrosion and fouling treatments are relatively easy to carry out [7].

The ESP system pump has its performance degraded in the presence of compressible gas. If the pump is operating on free gas, head deterioration, decreased efficiency and even gas locking can cause the production system to stop and shorten the life of downhole equipment. Environments that have their production affected by issues such as gas high-volume wells, produced solids, high temperature and corrosive environments often lead to multiple incidents of failure in ESP systems. This results in production losses and high operating costs, requiring workover [8].

Despite the natural separation in the annular space or the operation of a gas separator, the gas can eventually reach the pump, which will cause a significant reduction in its efficiency [9]. In addition, slight cyclical changes in the motor load and electric current arise in the pump, which significantly damages the ESP unit. Frequent system shutdowns and restarts eventually damage the motor and the entire installation run; pump life is severely reduced.

It is important to note that the gas inside the pump may be present in low or high amounts. If the gas volume is considered low, the system is not harmed. Often the amount of gas is so low that experts consider the operation to be normal gas operation. If the amount of gas is high, gas interference can occur, which significantly reduces the pump efficiency or gas locking that can interrupt the production of the well and, consequently, the pump cannot drain the fluid inside it.

According to this whole context, the general objective of this work is to use a Machine Learning (ML) classification algorithm to diagnose four operating conditions: normal operation, normal operation with gas, gas interference and gas locking of the ESP pump. This work has as its main motivation, the growing area of machine learning that will provide great changes in the industrial sector. In addition, ESP is an important method for artificial lift because it works with high liquid flow rates.

The ML algorithms used were Decision Tree, Support Vector Machine (SVM), K-Nearest Neighbor (KNN) and Multi-Layer Perceptron (MLP) neural network using the Python programming language in the Google Colaboratory^®^ (Google Colab) environment. For the operational diagnosis, real data from 24 wells that operate by electrical submersible pump in the region of Mossoró/RN, Brazil were used, the variable used in these wells was the electric current in a period of 24 h. The electric currents were classified by specialists in the oil area and distributed in the four operating conditions mentioned above.

The tests carried out in the present work used ML algorithms, with and without hyperparameter tuning, with application of an algorithm for unbalanced data and evaluation metrics. The complexity of this work is based on the similarity of the gas operation and gas interference charts, the difference between them is the range of the electric current. This situation causes difficulties in interpreting the algorithms. Despite this, the results of this research show that the ML algorithms used in this work were able to identify the four operating conditions of the ESP pump. The diagnostics obtained will be useful to identify the causes of the failures and, consequently, the field engineers will be able to apply effective solutions.

## 2. Theoretical Background

In this section, the concepts of machine learning classification algorithms that were used in this article will be discussed. Parameters that were used to improve the performance of the classification algorithms and the metric evaluated to observe how the algorithms behaved will be highlighted.

### 2.1. Classification Algorithms

In this work, supervised learning by the classification method was applied, as the classification task predicts discrete responses. It is recommended that data can be categorized, labeled or separated into specific groups or classes. Classification models identify input data into categories. In this article, the classification algorithms used were the Decision Tree, KNN, SVM and Multi-Layer Perceptron Neural Network (MLP) for comparison purposes, since they are different classification methods.

The Decision Tree (DT) algorithms are most commonly used in classification, as it is a technique that provides easily understandable modeling and simplifies the classification process [10]. The Decision Tree is a transparent mechanism that makes it easy for users to follow a tree structure to see how the decision is made. The central objective of the tree is to produce a model that calculates the value of a required variable based on numerous input variables [11].

Another well-known classification algorithm is the K-Nearest Neighbor (KNN), which classifies unlabeled observations, assigning them to the class of the most similar labeled examples. The characteristics of the observations are collected for the training and testing datasets [12]. An important concept that must be realized when applying KNN is the k-value relationship. To identify how many nearest neighbors there are around an analyzed sample, the value of k is measured. KNN assigns the label class to most of the closest K patterns in the data space [13].

The Support Vector Machine (SVM) was proposed by mathematician Vladimir Vapnik and it is based on a statistical theory of learning. SVM is considered one of the most prominent and convenient techniques to solve problems related to data classification [14] learning and prediction [15]. The classification performed in the SVM algorithm transforms the original training data into multidimensional data [16]. This classification technique employs the data vectors of a hyperplane in a huge dimensional space [17].

Finally, the Neural Network (NN) system consists of a series of interconnected processing elements, commonly called neurons. NNs receive information as inputs on one side and provide outputs on the other side using unidirectional connections between neurons in different layers [18]. The neural network Multi-Layer Perceptron (MLP) type has more than one perceptron, established in different layers. This makes it capable of solving non-linear problems.

Generally speaking, the applications of MLP’s are categorized as pattern classification, data prediction and function approximation. Pattern classification implies classifying data into predefined discrete classes, while forecasting refers to predicting future trends according to electric current and past data [19].

### 2.2. Classification Parameters

Classification parameters are techniques used for machine learning algorithms to be more assertive in their results. In this work, hyperparameters and the data balancing technique were used. In addition, the confusion matrix was applied to visualize the results, which also helped in the analysis of the results of the algorithms.

#### 2.2.1. Hyperparameter Tuning

Hyperparameters are essential for ML algorithms. For a set of training algorithms, with different hyperparameters, an ML algorithm can obtain models with significantly different performance from the test dataset [20]. Hyperparameters will help to increase the assertiveness of ML algorithms, the most common techniques for adjusting models with hyperparameters are Grid Search and Random Search [21].

One of the hyperparameter techniques that helps in greater assertiveness is the Grid Search, which divides the domain of hyperparameters into a discrete grid. Then, all combinations of values from this grid are tested, calculating some performance metrics using cross-validation [22]. The grid point that maximizes the average value in cross-validation is the optimal combination of values for the hyperparameters. The exposed methodology was used in this research.

#### 2.2.2. Unbalanced Dataset

Unbalanced datasets in ML result in data classification problems as the data is represented in an unequal way. In many real-world applications, the nature of the problem implies sometimes significant deviation in the class distribution of a binary or multiclass classification problem [23].

There are data-level methods that manipulate the training data, aiming to change the distribution of classes to a more balanced one. Techniques in this category add instances to minority classes through duplication or generation of new samples (oversampling) [24].

Oversampling replicates the minority class data until it is numerically equal to the majority class data [25]. In this work, due to the difference in distribution between the operating conditions, the oversampling technique was used, which randomly duplicates instances of minority classes to obtain a more balanced data distribution.

#### 2.2.3. Confusion Matrix

**Figure 2:**
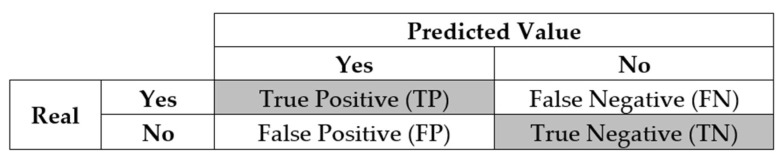
Confusion matrix for binary classification problems (adapted from [27]).

The confusion matrix is a very popular measure used in solving classification problems. It can be applied to binary classification and to multiclass classification problems [26]. An example of a confusion matrix for binary classification (2 × 2) is shown in Figure 2.

The “TN” output shows the number of accurately classified negative examples. Likewise, “TP” indicates the number of accurately classified positive examples. The term “FP” shows a false positive value, that is, the number of real negative examples classified as positive; and “FN” is characterized by the number of real positive examples classified as negative. One of the most commonly used metrics when performing classification is precision. The precision of a model (via a confusion matrix) is calculated using Equation (1). Precision is the positive cases correctly predicted by the classifier.

(1) $$precision=\frac{\mathrm{TP}}{\mathrm{TP}+\mathrm{FP}}$$

The recall metric is defined as the number of accurately ranked positives (TP) divided by the total number of predictions that are actually correct. The accuracy of an algorithm is represented as the sum of positive predictions (TP + TN) by the total number of predictions (TP + TN + FP + FN). Equations (2) and (3) show how the metrics of recall and accuracy are calculated.

(2) $$\;recall=\frac{\mathrm{TP}}{\mathrm{TP}+\mathrm{FN}}$$

(3) $$accuracy=\frac{\mathrm{TP}+\mathrm{TN}}{\mathrm{TP}+\mathrm{TN}+\mathrm{FP}+\mathrm{FN}}$$

Another metric that can be mentioned when mentioning the confusion matrix is the F1-score, which is the harmonic mean of precision and recall, as seen in Equation (4). All these parameters were calculated in the work in question.

(4) $$F_{1}score=\frac{2\;\left[\left(precision\right)\left(recall\right)\right]}{precision+recall}\;$$

## 3. Methodology

**Figure 3:**
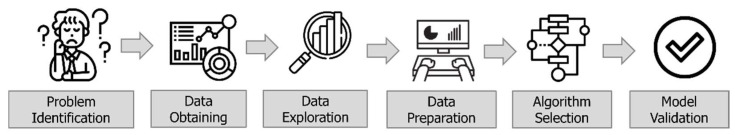
Common steps for trying out a machine learning project. These steps were used in the development of this work (adapted from [28]).

In this paper, the ML classification was divided into six steps: problem identification, data obtaining, data exploration, data preparation, algorithm selection and model validation. Initially, it is very important in ML classification to identify the focus of the problem to determine the feasibility of the procedure. The second step was to obtain the data. Subsequently, the exploration and preparation of the data were carried out so that, finally, the algorithm selected for this problem could be used. The last step of this classification process (model validation) was to analyze whether the results obtained were consistent from the validation of metrics. The steps mentioned are shown in Figure 3 [28].

### 3.1. Obtaining Data

The second step in ML experimentation is getting the data. In this step, it is very important to identify where the data is being collected. In addition, the correct choice of interface used for data manipulation is paramount. In this case, the Google Colaboratory^®^ (Colab) environment was used with the Python language. After these steps, the manual classification process began with the help of specialists.

**Figure 4:**
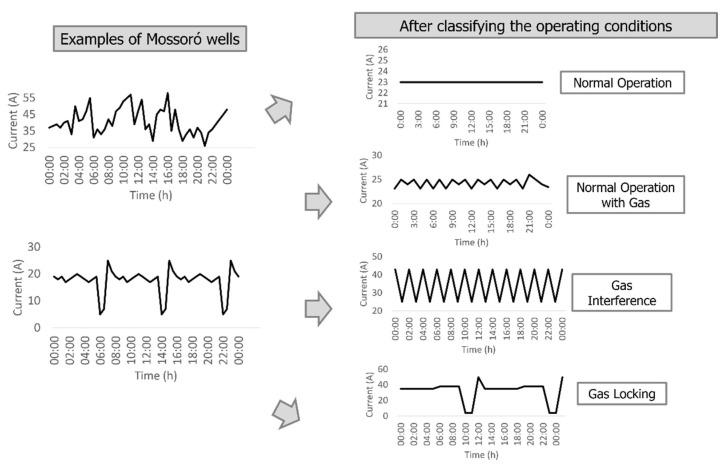
Classification scheme and manual selection of electric current data used in this work.

This work analyzed the behavior of 24 wells in Mossoró, RN, Brazil, however, 4 were discarded due to continuous motor shutdown. The classification was given from 20 wells to identify four types of operating conditions (Figure 4):

i. Normal Operation;
ii. Normal Operation with Gas;
iii. Gas Interference;
iv. Gas locking.

The operating conditions chosen were based on the gas variation within the ESP pump. The normal operation is the standard condition for ESP systems. Normal operation with gas was employed in this research, because the most ESP wells work with a small amount of gas inside the pump, but continue to work normally. To visualize more extreme conditions that could cause possible future pump stoppages, it was decided to work with gas interference and gas locking.

The identification was performed as follows: the dataset of the 20 wells were analyzed for 30 days and the specialist scanned these wells using an algorithm implemented in Python language. First, an algorithm was carried out to read the datasets of the 20 wells. After that, the specialist identified which well he wanted to analyze, and observed when the phenomenon began (day and time) and when it ended.

The developed algorithm showed the graph of electric current versus time of a well for 30 days and it was possible to select which time interval some operating condition was seen. To maintain a standard of identification of operating conditions, 24 h stretches were selected. The four operating conditions obtained were trained on a test dataset, using an ML algorithm.

**Figure 5:**
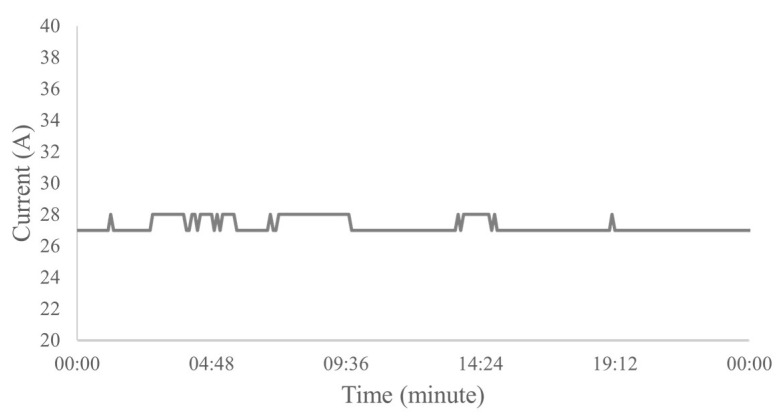
Example of a real well operating normally for 24 h.

The electric current versus time graph of the normal operating condition is smooth, however, this condition may produce a curve slightly above or below the ideal amperage of this operation, but this generated deviation from the curve does not prevent the system from working properly. According to engineers in the area, to classify normal operation, a constant pattern of electric current over time or peaks with variation of up to 1 A (Figure 5) was observed, which is considered a well with practically no electric current fluctuations.

**Figure 6:**
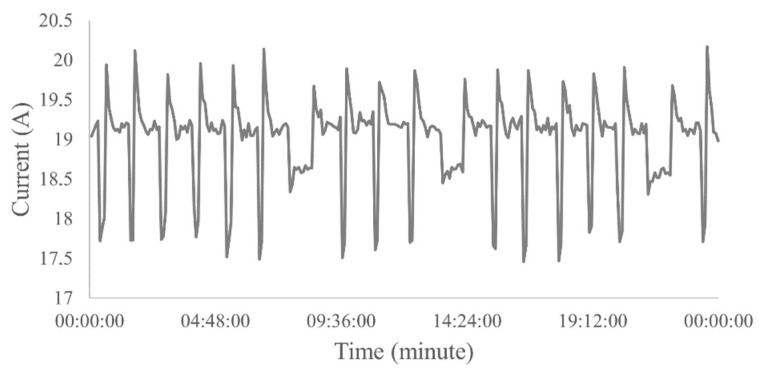
Example of a real well operating with gas for 24 h.

Normal gas operation was studied as it is a striking feature in the wells analyzed in this work. Therefore, according to engineers in the area, for wells with normal operation with gas, reference standards for electrical current ranging from 1 A to 5 A. This condition of the pump operating with a low amount of gas does not affect the production system, and the well disarm is not triggered. Figure 6 presents an example of a dataset of normal operation with gas used in the work.

**Figure 7:**
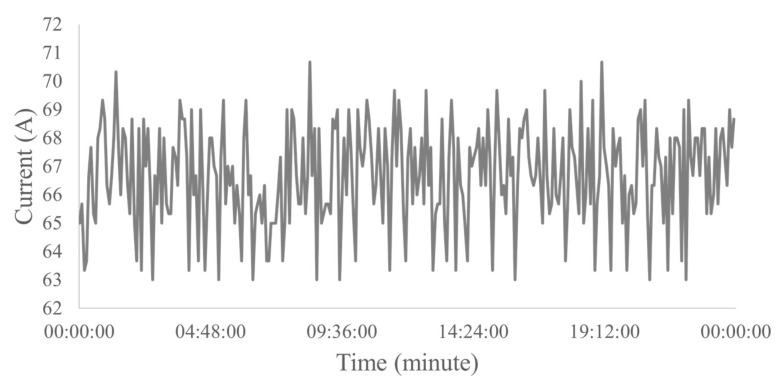
Example of a real well with gas interference for 24 h.

Gas interference (Figure 7) causes instability in pump operation, generating significant fluctuations in electric current. So, it is important to have an early diagnosis of what is happening in the system. It was considered for this paper, based in real wells, that ESP operations that had the variation of electric current above 5 A, would be considered gas interference.

**Figure 8:**
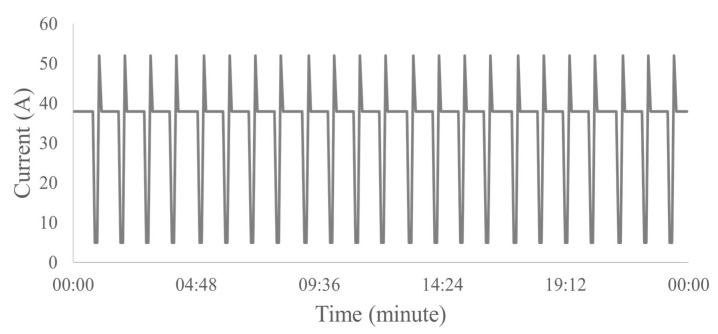
Example of a simulated 24 h gas locking well.

In the gas lock condition, characteristic illustrated in Figure 8, the electric current has a very peculiar characteristic. Initially, the electric current is constant until the beginning of a gas interference is visualized. The gas amount increases, and it became so high that the system stops, causing the system to disarm. As it is a very extreme fault, it is not easy to obtain from real well data. Therefore, in the data from the 20 analyzed wells, it was not possible to identify this problem. However, as it was necessary to obtain data for training and tests, datasets from simulated wells were used, based on reference [29].

### 3.2. Data Exploration

Exploring the data means doing a thorough analysis of every dataset obtained. Therefore, after classifying the expert, the data analysis in this work was based on viewing all datasets collected by the expert and separating them for each operating condition within a 24 h interval.

As previously stated, the gas locking phenomenon was not found in the wells, so 50 datasets were simulated so that the algorithm had data available to perform the ML algorithm tests. Table 1 shows the distribution of operating conditions that will be used for training in this work, in total there were 179 datasets.

### 3.3. Data Preparation

In data preparation, the information in the dataset is verified, that is, adjustments are made at this stage so that the quality of the model is not compromised. Some examples of adjustments are normalizations, standardizations, exclusion of outliers, among others. In addition, it is also at this stage that there is a separation of data into training and testing. The data preparation carried out in this work was initially based on the exclusion of some data from the wells, as there were long periods of pump downtime. Then, the data were interpolated so that they had the same number of points. Finally, the data were separated into training and testing.

#### 3.3.1. Linear Interpolation

Linear interpolation is a useful technique for fitting curves using linear polynomials. One method for estimating the value of a function between any two known values is called interpolation—Equation (5). In a given interval where there is a set of existing points, new points are created so that there are more points on a curve.

(5) $$\;\;\;y=y_{1}+\frac{\left(x-x_{1}\right)\left(y_{2}-y_{1}\right)}{x_{2}-x_{1}}$$

where $x_{1}$ and $y_{1}$ are the first coordinates; $x_{2}$ and $y_{2}$ are the second coordinates; x is the point to perform the interpolation and y the interpolated value. This formula is using coordinates of two given values to find the best fit curve as a straight line. So, this will give you whatever value of y is needed at a known value of x.

A standardization was applied in this work in order to leave all data with the same number of points. The dataset ranged from 159 to 344 points, as the largest dataset had 344 points, all other datasets were adjusted to this amount. This is necessary, so that the datasets have the same pattern. After standardization, the scale for each graph was from 0 to 75 A following [29].

#### 3.3.2. Test and Training Set

Before selecting the algorithm, it is very important to separate the data into test and training sets. The test set is the one that will be used as a basis for training the well data and its proportion is much smaller than the training set. In the data mining area, generally, 70% of the data are used for training and 30% for testing [30]. The proportion of 70–30 was the same applied in this work. Of the 179 datasets, 125 were defined as training and 54 as testing. Table 2 shows the organization of training and testing data, based on the datasets in Table 1.

### 3.4. Algorithm Selection

At the stage of algorithm selection, initially, no adjustment or manipulation was performed to improve the performance of the algorithm. Furthermore, in this work, algorithms with an increasing order of complexity were applied. The initial algorithms were executed without hyperparameter tuning or balanced data. After this test, hyperparameter tuning (Grid Search) and data balancing were applied, using the oversampling technique in the algorithms.

In the work in question, the order of choices of the algorithms was in order of complexity, the first to be tested was the Decision Tree (DT), then K-Nearest Neighbor (KNN), Support Vector Machine (SVM) and the Multi-Layer Perceptron (MLP) Neural Network. The choice of this set of algorithms, specifically, was in order to evaluate the distinct characteristics that each one can offer for this work. All models had hyperparameter conditions (Grid Search) evaluated. The hyperparameters implemented for each model are shown in Table 3.

In DT, the studied hyperparameters were: the function to measure the quality of a split in the tree (Criterion), strategy to choose the split of each node (Splitter), the minimum number of samples needed to be in a leaf node (Min_samples_leaf), the minimum number of samples needed to split an internal node (Min_samples_split) and the parameter to identify if there are any nodes with any faults or impurity (Max_leaf_nodes).

In KNN, the hyperparameters used were the Euclidean and Manhattan metrics (Metrics), the number of near neighbors used by default (Neighbors), the weight function used in the prediction (Weights), and the algorithm used to calculate the nearest neighbors (Algorithm). In SVM, the metrics studied were the regularization parameter (“C”), the kernel that specifies the type of kernel to be used in the algorithm and the coefficient applied to the kernel (gamma).

In the Neural Network, the activation function was applied to the hidden layer (Activation), the penalty parameter (Alpha), the Hidden_layer_sizes that represents the number of neurons of the hidden layer, the activation function to the hidden layer (Activation), the learning rate schedule for weight updates (Learning_rate), the initial learning rate used (Learning_rate_init), and the solver for weight optimization (Solver).

After the hyperparameter tuning step, the datasets had their data balanced by the random oversampling technique. This technique was used due to the duplication of the minority class examples in the training dataset. Thus, all datasets were adjusted to 53 train set according to the majority class of training data. The data were adjusted to this value, as it was the largest number of datasets obtained by the training set algorithm, so all should have the same amount of data.

## 4. Results

In the following topics, the results of this research will be exposed. The analysis of the metrics of the results will be carried out by applying hyperparameters, in addition to observing the behavior of unbalanced and balanced data for each chosen algorithm.

### 4.1. Decision Tree (DT)

Based on the scikit-learn library, the Decision Tree model was trained with the DecisionTreeClassifier function. This tree model is the simplest to interpret. In the tests of this model, three algorithms were implemented, the first without hyperparameter tuning and unbalanced data, a second with hyperparameter tuning and unbalanced data and the third with hyperparameter tuning and balanced data.

**Figure 9:**
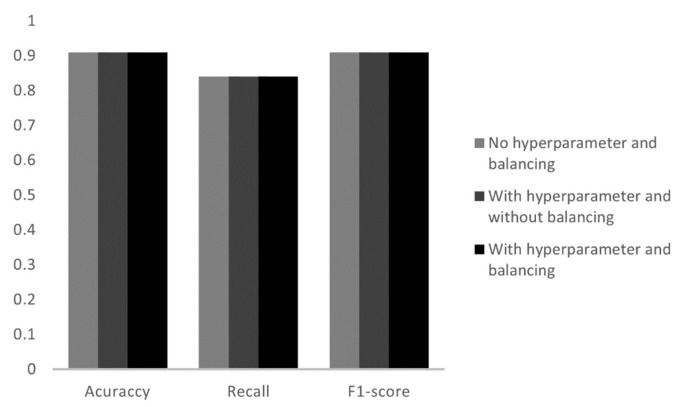
Analysis of the metrics of the three tests in the Decision Tree.

When applied to the Decision Tree algorithm, it was seen that all three models had the same result as shown in Figure 9. The accuracy value was 91%, recall 84% and F1-score 91%. The similar values in the parameters shows that, regardless of the hyperparameters, the proposed tree model will always have the same values of accuracy, recall and F1-score. Balancing the data, leaving everyone with the same amount of training datasets, did not affect the final result, since the random oversampling technique duplicates the existing datasets, so despite the greater number of training data, the behavior of the electric current would be the same as the existing datasets, which did not cause any difference in the final accuracy.

Although the result is similar in all cases, one of them was collected for the purpose of interpretation, and from that all test parameters (Table 4) can be analyzed.

In the table, the values of true positives (TP) are the total of data that the model got right, the true negatives (TN) are related to the sum of the correct data (diagonal of the matrix in bold) that were 49, minus the correct data of each condition. The false positives (FP) are the errors of the condition studied in the column, for example: two datasets of the normal condition were confused with the normal with gas, so there are two FP for the normal condition with gas. Finally, the false negatives (FN) are the errors of the condition studied in the line, in the normal condition with gas only one dataset is confused with the gas locking condition.

Analyzing what is exposed, it can be concluded that the highest number of hits were in the normal condition, despite the algorithm having two FN in this category. Another condition analyzed is gas locking which had 100% of hits, however its accuracy was 93% as in a dataset of the normal gas (FP) it was confused with the lock condition.

The third most assertive condition was the normal with gas, which hit five out of six datasets, however its accuracy was 71% thanks to the FP value. The condition where the Decision Tree model was least correct was gas interference. Of the five datasets, only three were successful in the condition and two were FN of the normal condition. The recall and the F1-score were impaired, only the accuracy was 100%, because there are no FP values.

### 4.2. K-Nearest Neighbor (KNN)

**Figure 10:**
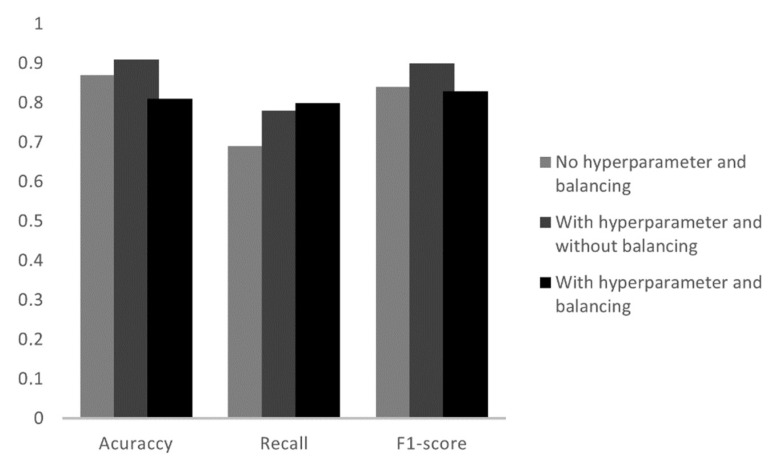
Analysis of the metrics of the three tests in the KNN model.

To train the data with the KNN model, from the scikit-learn library, the Kneigh-borsClassifier function was used. When testing this model, three algorithms were implemented. Initially, a simple model with the data still unbalanced and without hyperparameter adjustment, in this, the accuracy was 87%. Subsequently, hyperparameters were applied and the datasets remained unbalanced. It had the highest accuracy, 91%. The latter used hyperparameters and balanced data and had an accuracy of 81%. The criteria analyzed for each condition mentioned are shown in Figure 10. Table 5 shows all the parameters of the second algorithm.

The best result for the KNN model was with hyperparameters and without the data being balanced. Balancing the data in this algorithm had worse results than without balancing, in which case this method does not become applicable.

In the KNN model, in the same way as the DT, the normal condition and gas locking had the best results, the difference is that the KNN was able to set all the datasets of these two operating conditions right. However, there are five FP values for normal operation which lowers its accuracy to 86%. Of the six datasets, half were assertive in the normal category with gas and of the five with gas interference, three were recognized by the algorithm.

### 4.3. Support Vector Machine (SVM)

**Figure 11:**
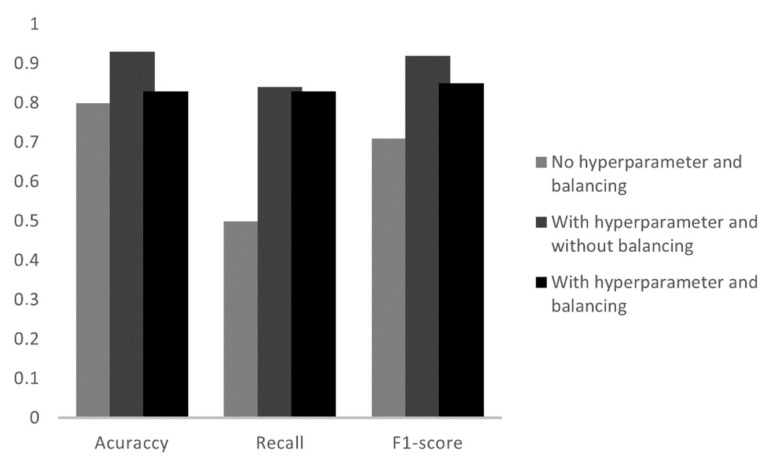
Analysis of the metrics of the three tests in the SVM model.

As in the previous models, the library used for training the datasets in the SVM algorithm was scikit-learn and the function applied was svm.SCV. In the tests of this model, three algorithms were performed. It started with a simple model with the data still unbalanced and without hyperparameter adjustment. In this case the accuracy was 80%. Later, the application with hyperparameters and with datasets unbalanced, had an accuracy of 93%. The latter used hyperparameters and balanced data and had an accuracy of 83%. The criteria analyzed for each condition mentioned are shown in Figure 11. Table 6 shows all the parameters of the algorithm with the best accuracy.

The application of hyperparameters increased the accuracy of the model without hyperparameter by 13 percentage points, however, when using the balanced dataset there is a decrease of 10 percentage points, going from 93% accuracy with hyperparameters and without balancing to 83% with hyperparameter and balancing. In this case, the balancing method is not applicable to the model in question.

The best result, observed in the table, was the normal operation that had a 100% recall. In gas locking, of the 13 datasets studied, the algorithm got 12 right, so it generated 1 FN. As in the gas locking, the number of FN was low, the recall and F1-score were 92% and 96%, respectively. As with the models mentioned above, the SVM reached the mark of three sets of correct data in the category of gas interference, which was five. This generated two FN’s that were confused by normal gas operation and normal operation. Finally, we have the normal operation with gas that hit six out of five datasets, the confusion was in the normal operation that generated 1 FN.

### 4.4. Neural Network (NN)

**Figure 12:**
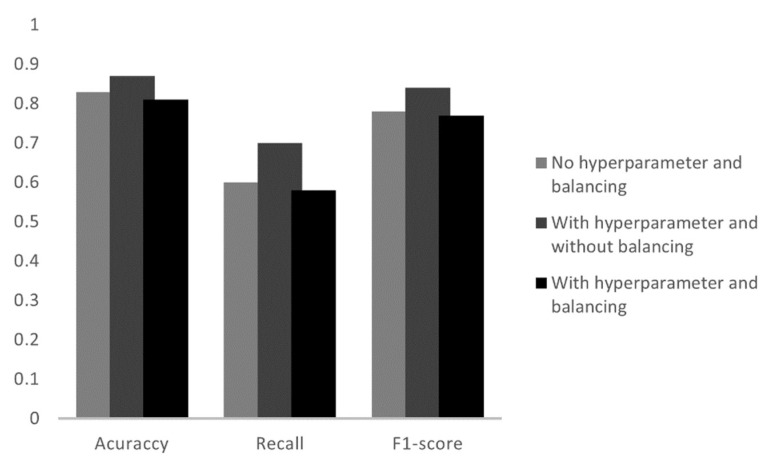
Analysis of the metrics of the three tests in the Neural Network.

To train the data with the MLP Neural Network, from the scikit-learn library, the MLPClassifier function was used. In the tests of this model, three algorithms were performed. The first was a simple model with the data unbalanced and without hyperparameter tuning. In this one, the accuracy was 83%. The second had the application of hyperparameters and the datasets remained unbalanced, with the highest accuracy, 87%. The latter used hyperparameters and balanced data and had an accuracy of 81%. The criteria analyzed for each condition mentioned are shown in Figure 12. Table 7 shows all the parameters of the algorithm with the best accuracy.

The best result for the NN model of the MLP type was with hyperparameters and without the data being balanced. Balancing the data in this algorithm had worse results than without balancing, in which case this method does not become applicable.

The Neural Network algorithm had the gas locking and normal operation with the best results, as seen in the KNN and SVM models, the difference is in the number of hits. In the case of NN, it generated one NF and five FP under normal operating conditions. In the gas locking, it got it right all with no FP and FN, achieving an accuracy, recall and F1-score of 100%. The Neural Network also excelled in the condition of normal operation with gas, the algorithm hit five of the six datasets generating only one FN.

However, the problem with this algorithm was the gas interference, it could not identify any of the five datasets. The algorithm confused the electric current behavior with normal operation and normal operation with gas, generating five FN. It also had one FP which was obtained thanks to the dataset of normal operation. Therefore, the precision, recall and F1-score of this condition was 0% causing a sharp drop in the model’s accuracy.

### 4.5. Limitations of the Presented Methods

For the 179 datasets analyzed there are several that are visibly similar, such as some of the datasets of normal with gas and gas interference. What really differentiates each operating condition is the variation of the electrical current amplitude, as mentioned in Section 3.1. Therefore, to improve the accuracy of each system, after the interpolation a scale from 0 to 75 A was used so that, in addition to the visual conditions of each graph, it was possible to identify the amplitude of the variation of the electric current.

Even so, the algorithms still cannot differentiate the datasets from the conditions mentioned above, as some still behave very similarly. This limitation is seen in the confusion matrix data, causing a decrease in the accuracy of the methods.

## 5. Discussion

The analysis of the confusion matrix presents the results of the classification algorithms in a detailed way, however, they are not enough to prove that the experimental results are significant. To validate the results obtained in Section 4, tests of variation of means were used, these were: the analysis of variance test (ANOVA) [31] followed by the Tukey’s test.

**Figure 13:**
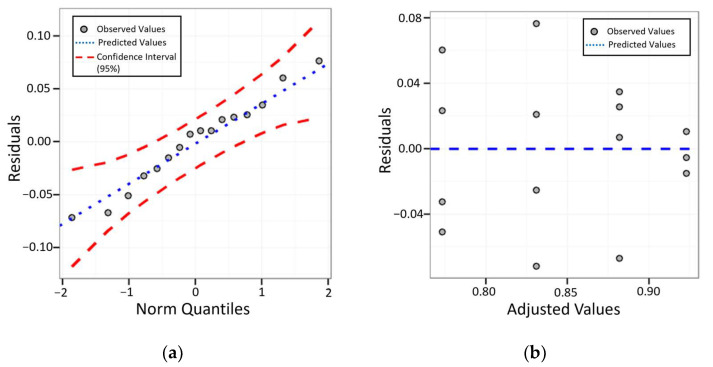
Residue analysis. (**a**) Comparison diagram of normal quantiles for model residuals; (**b**) Graph of residuals versus adjusted values.

The tests were performed using the Action Stat statistical software. For the tests, four replications were performed for each model. It is important to emphasize that the guarantee of the reliability of the statistical model is only possible when the ANOVA assumptions are met. In this work, this was confirmed with tests of normality and variability of the residuals. This can be seen in Figure 13.

It is possible to analyze in Figure 13a that the residuals are normally distributed according to the Anderson–Darling normality test (n = 16; AD = 0.201 and *p*-value = 0.856). Figure 13b proves that the residuals are well distributed along the zero axis, declaring that the data are acceptable for using ANOVA. In ANOVA, the *p*-value was verified, which must be less than or equal to the 5% significance level. In the case of this work, the *p*-value = 0.00428. Therefore, it is concluded that there is a high correlation between the data of this article and this value is very strongly against the null hypothesis.

With the validation of the *p*-value rejecting the null hypothesis, the Tukey’s test was performed to test whether there were differences between the variables, which in this case were classification algorithms (DT, KNN, SVM and NN). The results of this analysis are shown in Table 8.

**Figure 14:**
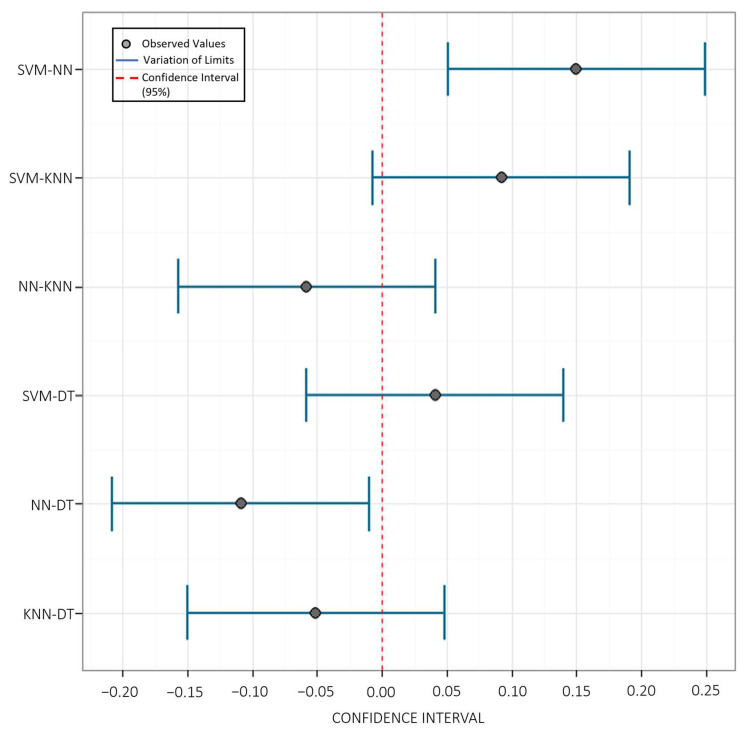
Differences between classification algorithms (DT, KNN, SVM and NN) based on confidence interval (95%).

Table 8 presents the mean of the replicates of each algorithm based on groups “a” and “b”. The best result of the averages was from the Support Vector Machine algorithm. Furthermore, the result confirms that the SVM and DT algorithms differ from the NN algorithm by Tukey’s test at 5% probability. The KNN algorithm does not differ from any tested model. To make it more visual, Figure 14 confirms what was seen in Table 8. All combinations of algorithms that cross the red dashed line are related to each other. The *p*-value of the SVM-NN was 0.004, showing that among the algorithms tested in this work, the Support Vector Machine and the Neural Network are the ones that most differ from each other, as their *p*-value is the furthest from 5% of the range reliable. The algorithms that have the highest relationship are the Support Vector Machine and the Decision Tree with a *p*-value of 0.621.

After statistical analysis, it was found that the four models analyzed can be applied to identify operating conditions in ESP pumps. The best results were with the SVM algorithm due to the support vectors formed by the algorithm that show the data closest to the decision surface. Therefore, there is greater precision when dealing with similar datasets. However, the NN had a lower accuracy than the other algorithms, as its accuracy in the gas interference condition was lower. This can be explained because for the NN the amount of input data for testing was low so that it was possible to make the necessary combinations in the neurons.

As can be seen in the results of the confusion matrices, the best precision was for the gas locking condition. This can be explained, because such a condition had its dataset simulated, in this way, the characteristics are specific and the algorithm can identify them assertively. Normal operation also has a high level of precision, as its graphs are more linear or with very small peaks, so there is no great difficulty for ML algorithms to identify them.

Hyperparameter analyses increased the metrics of almost all models, leaving just the same ones in the Decision Tree. However, the application to balance the training set was not an effective method for this work.

## 6. Conclusions

This work presented a solution in the machine learning branch to provide a diagnosis of operating conditions in electrical submersible pumps. The conditions studied were normal operation, normal operation with gas, gas interference and gas locking. The proposed study analyzed real data in the interior region of the state of Rio Grande do Norte and simulated data for the gas locking condition.

The four operating conditions mentioned were classified, so that the data were standardized by the interpolation method and, finally, the data were divided into 70% training and 30% testing. The validation procedure considered different classifiers, Decision Tree (DT), Support Vector Machine (SVM), K-Nearest Neighbor (KNN), and Multi-Layer Perceptron (MLP) Neural Network and a statistical study helped in the selection of the best classifier for this work.

The use of hyperparameters in most cases helped to improve the accuracy of the system. Regarding the equilibrium state of the training datasets, no major differences were observed in the results. This is explained by the duplication of training data that must have generated confusion in the algorithm, impairing the correct identification of the datasets, since most of the graphs are similar to each other and what is changed is the amplitude of the electric current.

The results showed that any of the evaluated classifiers can solve the problem and facilitate the work of a field engineer and, consequently, reduce the time spent in the process of diagnosing pump operating conditions. It is worth mentioning that for the gas interference condition the Neural Network had the lowest precision. The models that produced an accuracy rate greater than 90% were the Decision Tree, the KNN model and SVM. The best accuracy was the SVM algorithm with 93%, recall 84% and F1-score 92%. The hyperparameters used in the SVM model were gamma = 0.0001, kernel = rbf, and C = 10.

Data analysis by machine learning is growing nowadays, and in this work it was possible to identify operating conditions of the ESP system with a high level of assertiveness. In addition, the parameter used to identify the conditions of the aforementioned lift method was the electric current, which has a noisy and often unstable signal. The use of this parameter made the work innovative.

As future work, it is recommended to evaluate more operating conditions such as sand influence, insufficient well flow, frequent stopping and restarting of the pump, frequent load change due to emulsification, among others. In addition, it is indicated to analyze other variables that would assist the electric current, such as frequency, pump suction pressure and gas oil ratio (GOR).

## Figures and Tables

**Table 1:** Distribution of operating conditions.

Types of Operating Conditions	Number of Events in the Collected Data
1—Normal Operation with Gas	28
2—Gas Interference	20
3—Normal Operation	83
4—Gas Locking	50

**Table 2:** Distribution of testing and training data.

Types of Operating Conditions	Test Data	Training Data
1—Normal Operation with Gas	6	22
2—Gas Interference	5	13
3—Normal Operation	30	53
4—Gas Locking	13	37

**Table 3:** Hyperparameters of each model based on the scikit learn library.

Method	Hyperparameters	Conditions
DT	Criterion	Gini and Entropy
	Splitter	Best and Random
	Min_samples_leaf	{1; 2; 3; 4}
	Min_samples_split	{2; 3; 4}
	Max_leaf_nodes	{2 the 100}
KNN	Metrics	Euclidean and Manhattan
	Neighbors	{1 the 16}
	Weights	Uniform, Distance and Callable
	Algorithm	
SVM	C	{0.1; 1; 10; 100; 1000}
	Gamma	{1; 0.1; 0.01; 0.001; 0.0001}
	Kernel	rbf
NN	Activation	Tanh and Relu
	Alpha	{0.0001; 0.05}
	Hidden_layer_sizes	{(50; 50; 50), (50; 100; 50), (100,)}
	Learning_rate	Constant, Adaptive and Invscaling
	Learning_rate_init	{0.0001; 0.05}
	Solver	Sgd and Adam

**Table 4:** Decision Tree parameters with hyperparameters and with unbalanced data.

	Normal with Gas	Gas Interference	Normal	Gas Locking	Recall	Precision	F1-Score	Total Data	TP	TN	FP	FN
Normal with Gas	**5**	0	0	1	83%	71%	77%	6	5	44	2	1
Gas Interference	0	**3**	2	0	60%	100%	75%	5	3	46	0	2
Normal	2	0	**28**	0	93%	93%	93%	30	28	21	2	2
Gas Locking	0	0	0	**13**	100%	93%	96%	13	13	36	1	0

**Table 5:** KNN parameters with hyperparameters and with unbalanced data.

	Normal with Gas	Gas Interference	Normal	Gas Locking	Recall	Precision	F1-Score	Total Data	TP	TN	FP	FN
Normal with Gas	**3**	0	3	0	50%	100%	67%	6	3	46	0	3
Gas Interference	0	**3**	2	0	60%	100%	75%	5	3	46	0	2
Normal	0	0	**30**	0	100%	86%	92%	30	30	19	5	0
Gas Locking	0	0	0	**13**	100%	100%	100%	13	13	36	0	0

**Table 6:** SVM parameters with hyperparameters and with unbalanced data.

	Normal with Gas	Gas Interference	Normal	Gas Locking	Recall	Precision	F1-Score	Total Data	TP	TN	FP	FN
Normal with Gas	**5**	0	1	0	83%	71%	77%	6	5	44	2	1
Gas Interference	1	**3**	1	0	60%	100%	75%	5	3	46	0	2
Normal	0	0	**30**	0	100%	94%	97%	30	30	19	2	0
Gas Locking	1	0	0	**12**	92%	100%	96%	13	12	37	0	1

**Table 7:** Parameters of the Neural Network with hyperparameters and with unbalanced data.

	Normal with Gas	Gas Interference	Normal	Gas Locking	Recall	Precision	F1-Score	Total Data	TP	TN	FP	FN
Normal with Gas	**5**	0	1	0	83%	83%	83%	6	5	44	1	1
Gas Interference	1	**0**	4	0	0%	0%	0%	5	0	49	1	5
Normal	0	1	**29**	0	97%	85%	91%	30	29	20	5	1
Gas Locking	0	0	0	**13**	100%	100%	100%	13	13	36	0	0

**Table 8:** Results of Tukey’s test analysis.

DT	KNN	SVM	NN
0.882 ^a^	0.831 ^ab^	0.923 ^a^	0.773 ^b^

^a,b^ Means followed by the same letters do not differ from each other at the level and 5%.

## Data Availability

The data present in this study are available on request from the J.B. author.

## References

[1] Albar A., Asfoor H., Goz A., Ansari N. Combining the power of IoT and big data to unleash the potential of digital oil field. Proceedings of the International Petroleum Technology Conference.

[2] Ratcliff D., Gomez C., Cetkovic I., Madogwe O. Maximizing oil production and increasing ESP run life in a brownfield using real-time ESP monitoring and optimization software: Rockies field case study. Proceedings of the SPE Annual Technical Conference and Exhibition.

[3] Gindy M.E., Abdelmotaal H., Botros K., Ginawi I., Sayed E., Edris T. Monitoring & Surveillance Improve ESP Operation and Reduce Workover Frequency. Proceedings of the Abu Dhabi International Petroleum Exhibition and Conference.

[4] Takács G. (2009). Electrical Submersible Pumps Manual: Design, Operations, and Maintenance.

[5] Bates R., Cosad C., Fielder L., Kosmala A. (2004). Taking the Pulse of Producing Wells—ESP Surveillance. Oilfield Rev..

[6] Oliva G.B., Galvão H.L., dos Santos D.P., Maitelli A.L., Costa R.O., Maitelli C.W. Gas Effect in ESP System Stage by Stage Analysis. Proceedings of the SPE Artificial Lift Conference—Latin America and Caribbean, Salvador.

[7] Al-Bimani A.S., Armacanqui S., Al-Barwani B., Al-Hajri S., Sipra I., Al-Riyami H. Electrical Submersible Pumping System: Striving for Sustainable Run-Life Improvement in Oman Oil Fields. Proceedings of the IPTC 2008: International Petroleum Technology Conference.

[8] Gupta S., Saputelli L., Nikolaou M. Applying big data analytics to detect, diagnose, and prevent impending failures in electric submersible pumps. Proceedings of the SPE Annual Technical Conference and Exhibition.

[9] Barbosa T.S. (2011). Ambiente para Avaliação de Controladores Fuzzy Aplicados ao Método de Elevação Artificial por Bombeio Centrífugo Submerso. Master’s Thesis.

[10] Brodley C.E., Utgoff P.E. (1992). Multivariate versus Univariate Decision Trees.

[11] Kesavaraj G., Sukumaran S. A study on classification techniques in data mining. Proceedings of the 2013 Fourth International Conference on Computing, Communications and Networking Technologies (ICCCNT).

[12] Zhang Z. (2016). Introduction to machine learning: K-nearest neighbors. Ann. Transl. Med..

[13] Soofi A.A., Awan A. (2017). Classification techniques in machine learning: Applications and issues. J. Basic Appl. Sci..

[14] Nizar A.H., Dong Z.Y., Wang Y. (2008). Power utility nontechnical loss analysis with extreme learning machine method. IEEE Trans. Power Syst..

[15] Xiao H., Peng F., Wang L., Li H. Ad hoc-based feature selection and support vector machine classifier for intrusion detection. Proceedings of the 2007 IEEE International Conference on Grey Systems and Intelligent Services.

[16] Chandra M.A., Bedi S.S. (2021). Survey on SVM and their application in image classification. Int. J. Inf. Technol..

[17] Ahmad I., Abdulah A.B., Alghamdi A.S., Kim T., Adeli H. (2010). Towards the designing of a robust intrusion detection system through an optimized advancement of neural networks. Advances in Computer Science and Information Technology.

[18] Goh A.T. (1995). Back-propagation neural networks for modeling complex systems. Artif. Intell. Eng..

[19] Guo Z.X., Wong W.K., Li M. (2012). Sparsely connected neural network-based time series forecasting. Inf. Sci..

[20] Wang B., Gong N.Z. Stealing hyperparameters in machine learning. Proceedings of the 2018 IEEE Symposium on Security and Privacy (SP).

[21] Mantovani R.G., Rossi A.L.D., Vanschoren J., Bischl B., de Carvalho A.C.P.L.F. Effectiveness of random search in SVM hyper-parameter tuning. Proceedings of the 2015 International Joint Conference on Neural Networks (IJCNN).

[22] Ghawi R., Pfeffer J. (2019). Efficient hyperparameter tuning with grid search for text categorization using KNN approach with BM25 similarity. Open Comput. Sci..

[23] Chawla N.V., Bowyer K.W., Hall L.O., Kegelmeyer W.P. (2002). SMOTE: Synthetic minority over-sampling technique. J. Artif. Intell. Res..

[24] Galar M., Fernandez A., Barrenechea E., Bustince H., Herrera F. (2012). A review on ensembles for the class imbalance problem: Bagging-, boosting-, and hybrid-based approaches. IEEE Trans. Syst. Man Cybern. Part C Appl. Rev..

[25] Weiss G.M., Mccarthy K., Zabar B. Cost-sensitive learning vs. sampling: Which is best for handling unbalanced classes with unequal error costs?. Proceedings of the International Conference on Data Mining.

[26] Kulkarni A., Chong D., Batarseh F.A. (2020). Foundations of data imbalance and solutions for a data democracy. Data Democracy.

[27] De Castro L.N., Ferrari D.G. (2017). Introdução a Mineração de Dados.

[28] Nascimento J., Maitelli A., Maitelli C., Cavalcanti A. (2021). Diagnostic of Operation Conditions and Sensor Faults Using Machine Learning in Sucker-Rod Pumping Wells. Sensors.

[29] Han G., Chen M., Zhang H., Ling K. Real-Time Monitoring and Diagnosis of Electrical Submersible Pump. Proceedings of the SPE Annual Technical Conference and Exhibition.

[30] Géron A. (2017). Hands-on machine learning with Scikit-Learn and TensorFlow: Concepts, tools, and techniques to build intelligent systems.

[31] Sthle L., Wold S. (1989). Analysis of variance (ANOVA). Chemom. Intell. Lab. Syst..

